# Iris classification based on sparse representations using on-line dictionary learning for large-scale de-duplication applications

**DOI:** 10.1186/s40064-015-0971-1

**Published:** 2015-05-23

**Authors:** Pattabhi Ramaiah Nalla, Krishna Mohan Chalavadi

**Affiliations:** Department of Computer Science and Engineering, Indian Institute of Technology Hyderabad ODF Estate, Medak, Telangana, 502205 India

**Keywords:** De-duplication, Biometrics, Iris fibers, Iris classification, Iris adjudication, Sparse representation, On-line dictionary learning

## Abstract

De-duplication of biometrics is not scalable when the number of people to be enrolled into the biometric system runs into billions, while creating a unique identity for every person. In this paper, we propose an iris classification based on sparse representation of log-gabor wavelet features using on-line dictionary learning (ODL) for large-scale de-duplication applications. Three different iris classes based on iris fiber structures, namely, stream, flower, jewel and shaker, are used for faster retrieval of identities. Also, an iris adjudication process is illustrated by comparing the matched iris-pair images side-by-side to make the decision on the identification score using color coding. Iris classification and adjudication are included in iris de-duplication architecture to speed-up the identification process and to reduce the identification errors. The efficacy of the proposed classification approach is demonstrated on the standard iris database, UPOL.

## Introduction

Various government sectors in the world provide welfare services like NREGS (national rural employment guarantee system), TPDS (targeted public distribution system), old age pensions, health insurance etc... for the benefit of the people. A unique identity (UID) number creation for every person removes the requirement of producing mutliple documentary proofs for availing the services. De-duplication of biometrics plays a key role in providing unique identity of a person. De-duplication means the elimination of duplicate enrollments of the same person using the biometric data. As the number of people enrolled into the biometric system runs into billions, the time complexity increases in the de-duplication process while creating a unique identity for every individual. There is a need for de-duplication architecture based on biometrics which are scalable in large-scale databases. Among all the biometrics, fingerprints and iris give more accurate results in uniquely identifying the people based on minutia features. The biometric recognition system allows few errors in the identification process. In order to reduce the errors, fingerprint experts look for possible fingerprint matches and enhance the fingerprints to compare the minutia features manually using fingerprint adjudication process. Fingerprint adjudication means, comparison of two fingerprints side-by-side to analyze the matched minutia features. Even though the iris biometric is more accurate than the fingerprints, there is a need for iris adjudication process to reduce the identification errors.

The complex iris texture provides the uniqueness for iris images. Daugman proposed an iris recognition system by using gabor filters and iris codes (Daugman 1993, 2001, 2003, 2004). Wildes 1997 has implemented a gradient iris segmentation using Laplacian pyramid construction. Few researchers already explored iris classification techniques based on hierarchical visual codebook (Sun et al. 2014), block-wise texture analysis (Ross and Sunder 2010) and color information (Zhang et al. 2012). There are no approaches for classification of iris images based on the pre-defined iris classes in the existing work. In this paper, we propose an iris classification based on sparse representation of log-gabor wavelet features using on-line dictionary learning (ODL). Three different iris classes based on iris fiber structures, namely, stream, flower, jewel and shaker, are used for faster retrieval of identities in large-scale de-duplication applications. Also, an iris adjudication process is proposed by comparing the matched iris-pair images side-by-side to make the decision on the identification score using color coding. The iris classification and adjudication framework is used to speed-up the identification process and to reduce the identification errors in iris de-duplication architecture.

The rest of the paper is organized as follows: Section 2, gives the details of sparse representation and on-line dictionary learning. Section 3, gives the motivation for the proposed iris classification approach by illustrating the complexity involved in de-duplication of large scale iris databases. In Section 4, the proposed iris classification and adjudication framework is presented. Experimental results of the proposed classification and adjudication framework are given in Section 5. Conclusions are explained in Section 6.

## Sparse representation and on-line dictionary learning (ODL)

Sparse representation has received a lot of attention from researchers in signal and image processing. Sparse coding involves the representation of an image as a linear combination of some atoms in a dictionary (Ramirez et al. 2010). Several algorithms like on-line dictionary learning (ODL) (Mairal et al. 2009), *K*-SVD (Aharon et al. 2006) and method of optimal directions (MOD) (Engan et al. 1999) have been developed to process training data. Sparse representation is used to match the input query image with the appropriate class. Etemand and Chellappa (Etemad and Chellappa 1998) proposed a feature extraction method for classification using wavelet packets. In (Sprechmann and Sapiro 2010), a method presented for the learning of dictionaries simultaneously. Recently, similar algorithms for simultaneous sparse signal representation have also been proposed (Huang and Aviyente 2006; Rodriguez and Sapiro^2008^).

The online dictionary learning algorithm alternates between sparse coding and dictionary update steps. Several efficient pursuit algorithms have been proposed in the literature for sparse coding (Engan et al. 1999; Mallat and Zhang 1993). The simplest one is the ***l***_***1***_-lasso algorithm (Lee et al. 2007). Main advantage with ODL algorithm is its computational speed as it uses ***l***_***1***_-lasso algorithm for sparse representation.

## De-duplication architecture

De-duplication means the elimination of duplicate enrollments of the same person using the biometric data. During de-duplication process, matching the biometrics of a person is done against the biometrics of other persons to ensure that the same person is not enrolled more than once.

*3.0.1 Motivation behind this work* The state government of Andhrapradesh (Government of Andhra Pradesh, civil supplies department 2015) in India undertake the responsibility to identify the eligible households/beneficiaries and issue a ration card which enables them to avail the prescribed quantity of food grains and/or other commodities. The de-duplication was carried out for the ration cards using 52 million people iris codes to reduce the misuse of government subsidy. There are over 6.26 quadrillion (6,262,668,889,152,840) iris matches performed in de-centralized manner to remove duplicate enrollments in 61 days with high-end blade servers equipment which is not a scalable solution. This is the motivation for the proposed classification approach which reduces the search time drastically and provide the scalable de-duplication solutions.

**Figure 1:**
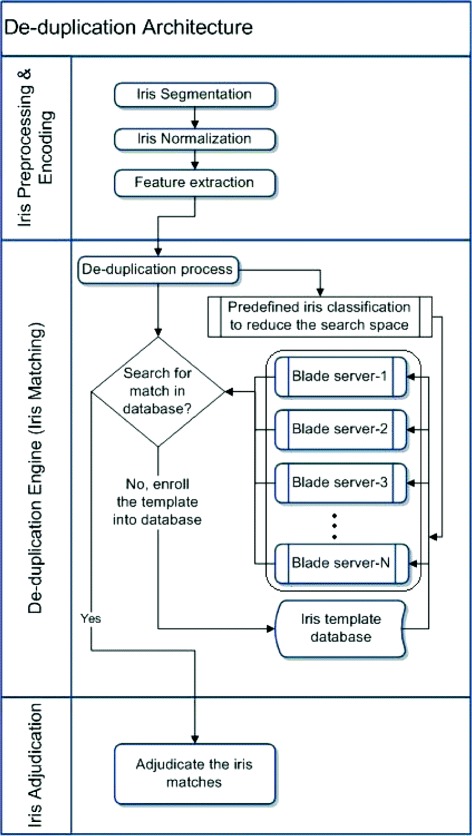
Iris de-duplication architecture.

The proposed de-duplication architecture is shown in Figure 1. In the processing stage, an iris image is segmented and normalized. Then iris templates are extracted using log-gabor wavelets. The de-duplication engine or iris matcher improves the speed of de-duplication by adding multiple blade servers. All the enrolled iris templates are loaded into each blade server and the iris templates are compared in *"*1:*all"* manner in *N* blade servers simultaneously. For example, if there are *N* query iris templates to be processed, then each query iris template goes to a blade server for de-duplication. If there are more than N query images, the delta of the iris templates keep on waiting in a queue till any of the blade servers are free. Increasing the blade servers is not an optimal solution, especially in large-scale iris databases. There should be another layer for iris classification to reduce the search space in the de-duplication engine. So, we propose an iris classification based on sparse representation of log-gabor wavelet features using on-line dictionary learning (ODL). Also, an iris adjudication process is done by comparing the matched iris-pair images side-by-side to know the confidence-level on the matching score based on color coding.

## Proposed iris classification and adjudication framework

**Figure 2:**
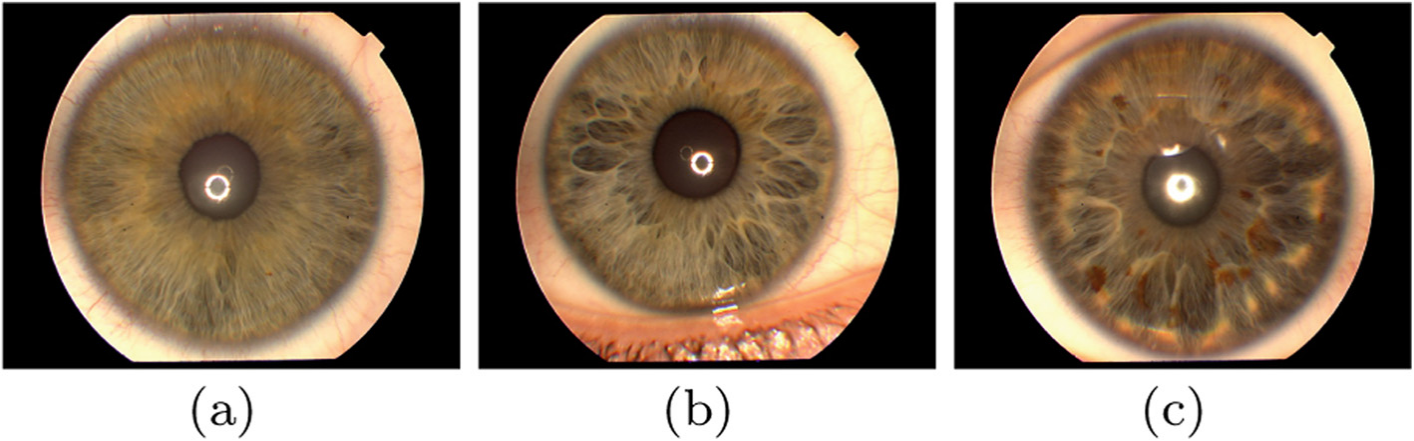
Iris classes: **(a)** stream, **(b)** flower and **(c)** jewel-shaker structures.

**Figure 3:**
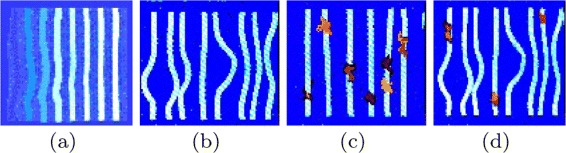
Iris fibers: **(a)** stream, **(b)** flower, **(c)** jewel and **(d)** shaker.

The proposed iris classification approach uses three different classes of iris images (Unitree foundation the rayid model of iris interpretation 2009) namely, stream, flower, and jewel-shaker as illustrated in Figure 2. The iris structure can be determined by the arrangement of white fibers radiating from the pupil. In stream iris structure, these fibers are arranged in regular and uniform fashion. The arrangement of fibers is irregular in the flower iris structure. In jewel iris structure, the fibers have some dots. The shaker iris structure have both the characteristics of flower and jewel iris structures. The arrangement of fibers are illustrated in Figure 3.

The following are the steps involved in the proposed iris classification and adjudication framework: ***Step 1.****Iris segmentation and normalization* : The pupillary and limbic boundaries (Masek 2003) of an iris image are approximated as circles using three parameters: the radius r, and the coordinates of the center of the circle, *x*_0_ and *y*_0_. The integrodifferential operator (Daugman 1993) used for iris segmentation is:

(1) $$  max(r,x_{0},y_{0}) G_{\sigma}(r)*\frac{\partial}{\partial r} \int\limits_{r,x_{0},y_{0}} \frac{I(x,y)}{2\Pi r}ds,  $$

where *G*_*σ*_(*r*) is a smoothing function and *I*(*x*,*y*) is the image of the eye.

**Figure 4:**
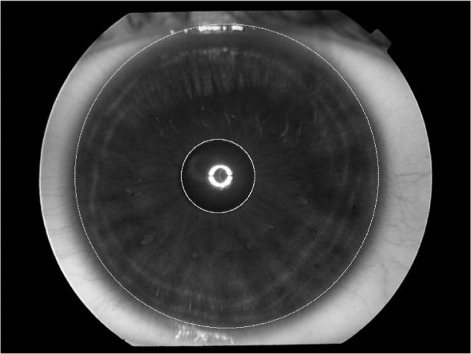
Iris image segmentation.

**Figure 5:**
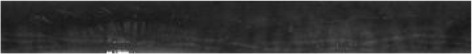
Normalized iris image.

After applying the operator, the resultant segmented iris image is as shown in Figure 4. The segmented iris image is then converted to a dimensionless polar coordinate system based on the Daugman Rubber Sheet model (Daugman 1993) as shown in Figure 5.

**Step 2.***Feature extraction*(Masek 2003): The log-gabor wavelet feature vector of size 720 × 40 is extracted from the normalized iris image of size 360 × 40. The resultant feature vector is converted to a single column vector by column major ordering. From each class, some of the iris images are selected to express as a linear weighted sum of the feature vectors in a dictionary belonging to three different classes of iris.***Step 3.****Iris classification using ODL*: An on-line dictionary learning (ODL) algorithm is used to classify the iris data into three different classes to reduce the search space. The weights associated with feature vectors in the dictionary are evaluated using ODL algorithm, which is a solution to *l*_1_ optimization for over-determined system of equations. The feature vectors which belong to a particular iris class carry significant weights which are non-zero maximum values. The class ***C***=[***C***_1_,…,***C***_*N*_] consists of training samples collected directly from the image of interest. In the proposed sparsity model, images belonging to the same class are assumed to lie approximately in a low dimensional subspace. Given *N* training classes, the ***p***^*th*^ class has ***K***_*p*_ training images $\{ \mathbf {y}^{N}_{i}\} \ i$=1,…, ***K***_*p*_. Let *b* be an image belonging to the ***p***^*th*^ class, and it is represented as a linear combination of these training samples:

(2) $$ b=\mathbf{D}^{p}{\Phi}^{p},  $$

where ***D***^*p*^ is a dictionary of size *m*×***K***^*p*^, whose columns are the training samples in the ***p***^*th*^ class and ***Φ***^*p*^ is a sparse vector.

The following are the steps involved in the proposed classification method:

1. *Dictionary Construction:* Construct the dictionary for each class of training images using on-line dictionary learning algorithm (Mairal et al. 2009). Then, the dictionaries ***D***=[***D***_1_,…,***D***_*N*_] are computed using the equation:
  $$  (\hat{\mathbf{D}}_{i},\hat{\Phi}_{i}) = \arg\min_{\mathbf{D}_{i},\Phi_{i}}\frac{1}{N}\sum_{i=1}^{N}\frac{1}{2}\left\|\mathbf{C}_{i}-\mathbf{D}_{i}\Phi_{i}\right\|_{2}^{2}+ \lambda \left\|\Phi_{i}\right\|_{1},  $$
  satisfying $ \mathbf {C}_{i} = \hat {\mathbf {D}}_{i} \hat {\Phi }_{i}, \quad i = 1,2,\ldots,N. $
2. *Classification:* In this classification process, the sparse vector ***Φ*** for given test image is found in the test dataset ***B***=[***b***_1_,…,***b***_*l*_]. Using the dictionaries of training samples ***D***=[***D***_1_,…,***D***_*N*_], the sparse representation ***Φ*** satisfying ***DΦ***= ***B*** is obtained by solving the following optimization problem:
  (3) $$  \Phi^{j} = \arg\min_{\Phi}\frac{1}{2} \|\mathbf{b_{j}}-\mathbf{D}\Phi_{j}\|_{2}^{2}\quad ;\  $$
  $ \textrm {subject to}\ \|\Phi _{j}\|_{1}\leq T_{1}, \ and \ \hat {i} = \arg \min _{i} \|\mathbf {b_{j}}-\mathbf {\mathbf {D}}\delta _{i}(\Phi ^{j})\|_{2}^{2},\ \;\;j=1,\cdots,t. $

where ***δ***_***i***_ is a characteristic function that selects the coefficients. Then ***b***_***j***_ is assigned to ***C***_***i***_ associated with the ***i***^***th***^ dictionary. It means, finding the sparsest dictionary for a given test data using ***l***_***1***_ -lasso algorithm. Then, test data is assigned to the class associated with this sparsest dictionary. **Step 4.***Iris Adjudication*: The matched iris pairs are compared using the adjudication process to illustrate the match-ability of iris images based on the similarity of iris regions marked with three different colors, namely, green, yellow and red. The green, yellow and red colors indicate good, poor and bad match, respectively. The normalized iris image is divided into different regions and the confidence-level of matching for each region is verifed and assigned a color code using the dissimilarity measurement.

## Experimental results

To enable the effective test of the proposed classification strategy, three standard iris image databases are used, namely, CASIA1 database (Casia-irisv1 chinese academy of sciencesinstitute of automation iris database 2015), IITD iris database (Kumar and Passi 2010, 2015), and UPOL iris database (Dobe and Machala 2004; Dobes̆ et al. 2006, 2004).

The CASIA database consists of 108 subjects, three instances of left iris and four instances of right iris are collected from each subject. So there is a total of 756 iris images in the database, all are having the image dimensions 320 × 280 gray-scale images. For testing, 216 iris images are used and the remaining iris images are used for training.

The IITD iris database consists of 224 subjects iris data, both left and right iris images. For each subject there are 10 instances of each iris image. So there is a total of 2232 iris images in the database, all are having the image dimensions 320 x 280 gray-scale images.

The UPOL iris data is collected from 64 subjects, with three samples of left and right eyes from each subject resulting in a total of 384 iris images. Each iris image is of 24 bit RGB color space with a high resolution image size, 768x576. The images were captured using the optical device (TOPCON TRC50IA) which is connected to a Sony DXC-950p 3CCD camera.

Experiments are performed using the following iris classification approaches: Approach-1: SVM-4Class-PCA-Kmeans Approach-2: ODL-4Class-PCA-Kmeans Approach-3: SVM-3Class-IrisFibers Approach-4: ODL-3Class-IrisFibers

The results are compared to demonstrate the efficacy of the proposed classification approach in the iris de-duplication architecture. The details are given below:

**SVM-4Class-PCA-Kmeans classification approach:**This classification approach uses the support vector machine (SVM) as a classifier. The classes are defined by applying the k-means clustering on the iris feature vectors whose dimensions are reduced to 100 features by considering the 100 principle components using principle component analysis (PCA). The correlation similarity measure is used for clustering the iris data into four different iris categories. This approach is applied on three standard iris databases, where 2/3 of the each database is used for training and remaining data is used for testing.

**ODL-4Class-PCA-Kmeans classification approach**In this classification approach, the sparsity-based on-line dictionary learning (ODL) is used as a classifier. The k-means clustering is applied to define the classes on the iris feature vectors whose dimensions are reduced to 100 features by considering the 100 principle components using PCA. The correlation similarity measure is used for clustering the iris data into four different iris categories. This approach is applied on three standard iris databases, where 2/3 of the each database is used for training and remaining data is used for testing.

**SVM-3Class-IrisFibers classification approach**This classification approach uses SVM as a classifier. The classes are defined by manual labeling of three iris categories (Unitree foundation the rayid model of iris interpretation 2009) using the iris fiber structures. This approach is applied on UPOL standard iris database, where 2/3 of the database is used for training and remaining data is used for testing.

**ODL-3Class-IrisFibers classification approach**Sparsity-based on-line dictionary learning (ODL) is used in this iris classification approach. The proposed iris de-duplication architecture include this classification to reduce the search space. The classes are defined by manual labeling of three iris categories using the iris fiber structures. This approach is applied on UPOL standard iris database, where 2/3 of the database is used for training and remaining data is used for testing.

### 5.1 Description of the experiments

#### 5.1.1 Experiment-1

In iris classification approaches 1 and 2, the experiments are conducted using the three databases, namely, CASIA1, IITD and UPOL iris dabases with template sizes 480 by 20. Four classes are identified using k-means clustering algorithm using the correlation-based distance metric. Table 1 describes the details of the number of images in each class and in three different databases.

**Table 1:** **Iris classes defined based on k-means clustering and PCA**

**# of Images in**	**CASIA1**	**IITD**	**UPOL**
Class-1	196	525	81
Class-2	203	500	114
Class-3	196	595	69
Class-4	161	580	120

**Figure 6:**
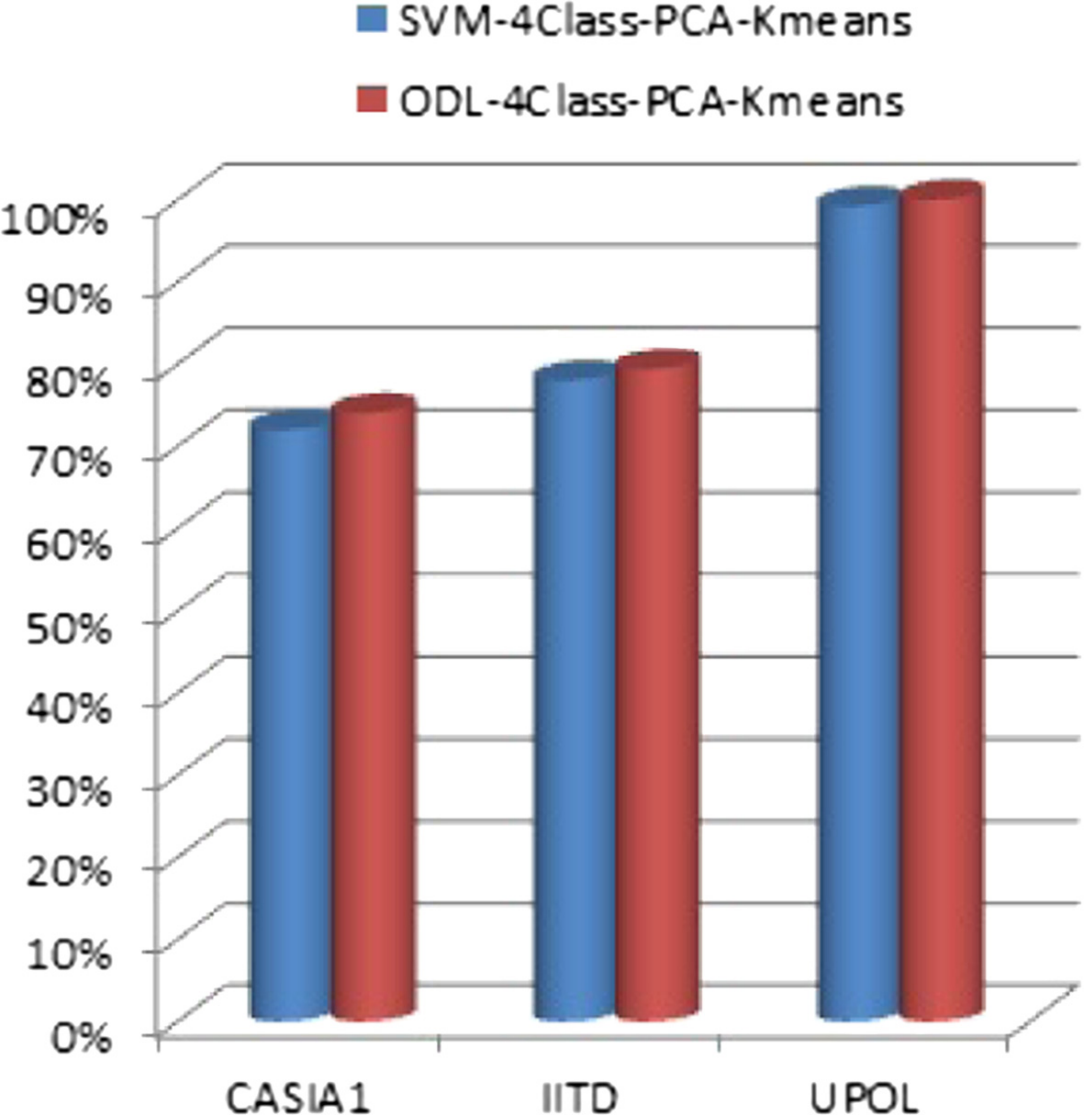
Experimental results for the classification approaches *SVM-4Class-PCA-Kmeans* and *ODL-4Class-PCA-Kmeans* for the three iris databases namely, CASIA1, IITD and UPOL.

The experimental results are illustrated as shown in Figure 6. It is observed that the *ODL-4Class-PCA-Kmeans* classification approach gives better classification performance due to the effectiveness of sparsity.

#### 5.1.2 Experiment-2

In iris classification approaches 3 and 4, the experiments are conducted using the UPOL iris database with template sizes 720 by 40. Three classes are manually identified in these proposed iris classification approaches using the iris patterns stream, flower and jewel-shaker as shown in Table 2. In this experiment, the other two databases are excluded as it was difficult to mark the class labels due to the less clarity to manually identify the iris fiber structures.

**Table 2:** **Iris classes defined based on the iris fibers stream, flower and jewel-shaker**

**Class**	**# of Images**	**Subject Ids**
	**(%)**	
Class-1	192 (50%)	001,006,007,008,011, 013,014,016,018,019,
(Stream)		020,021,023,024,026, 027,028,033,041,042,
		044,045,050,051,052, 053,058,059,060,061,
		062,064
Class-2	102 (26.56%)	002,009,010,015,017, 022,031,036,037,040,
(Flower)		043,047,048,049,054,
		056,063
Class-3	90 (23.44%)	003,004,005,012,025, 029,030,032,034,035,
(Jewel-Shaker)		038,039,046,055,057

**Figure 7:**
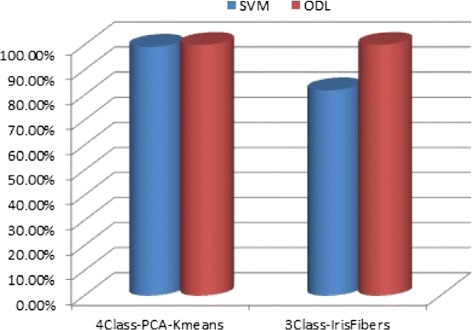
Experimental results for all the proposed classification approaches on UPOL iris database.

The experimental results for the UPOL database are compared using SVM and ODL and illustrated as shown in Figure 7. It is observed that the classification accuracy is better in the ODL-related classification approaches.

#### 5.1.3 Detailed analysis on the proposed classification approach : ODL-3Class-IrisFibers

In order to evaluate the performance of proposed classification approach using on-line dictionary learning, the database is split into three sets: training set, testing set and validation set. The distribution of all the three sets are taken in such a way that the 2 samples of each iris image is allotted to the training set and validation set, and the remaining iris sample is given to the test set. The training set consists of 224 images where 112 images are from Class-1 (Stream), 60 images are from Class-2 (Flower) and 52 images are from Class-3 (Jewel-Shaker). The number of test images selected from Class-1, Class-2 and Class-3 are 64, 34 and 30, respectively. A set of 32 iris images is assigned to validation set where 16 images belong to Class-1, 8 images belong to Class-2 and 8 images belong to Class-3.

The experiments were conducted in three different ways of choosing test sets (systematically selecting first, second or third samples of each iris) where the performance is almost similar. The classification performance is shown for the test data set with different dictionary sizes 60, 90 and 120, in Tables 3, 4 and 5, respectively.

**Table 3:** **Classification performance on test data set for dictionary size = 60**

**Class**	**Residual parameter**		
	0.5	**0.05**	0.005
Class-1 (Stream)	90.5	**97**	93.83
Class-2 (Flower)	91.18	**94.12**	88.2
Class-3 (Jewel-Shaker)	100	**100**	100

Boldface data represents the best performance.

**Table 4:** **Classification performance on test data set for dictionary size = 90**

**Class**	**Residual parameter**		
	0.5	**0.05**	0.005
Class-1 (Stream)	95	**100**	98.5
Class-2 (Flower)	94.12	**100**	97.06
Class-3 (Jewel-Shaker)	100	100	100

Boldface data represents the best performance.

**Table 5:** **Classification performance on test data set for dictionary size = 120**

**Class**	**Residual parameter**		
	0.5	**0.05**	0.005
Class-1 (Stream)	95	**100**	98.5
Class-2 (Flower)	91.18	**100**	96.06
Class-3 (Jewel-Shaker)	100	100	100

Boldface data represents the best performance.

**Figure 8:**
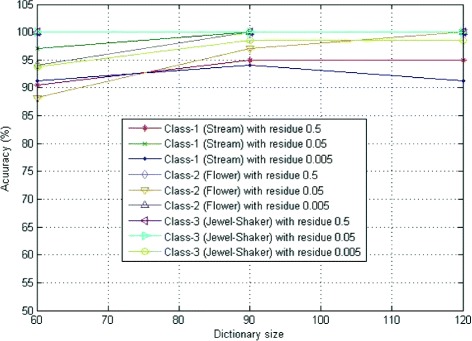
Classification accuracy for three different dictionary sizes 60, 90 and 120.

In Table 6, the classification accuracy for the validation data set is given. It is observed that 100% classification accuracy is achieved for the dictionary sizes, 90 and 120 with residual error value 0.05 as shown in Figure 8. The confusion matrix for both test data and validation data sets are shown in Table 7.

**Table 6:** **Classification performance on validation data set for dictionary sizes 60, 90 and 120**

**Class**	**Dictionary sizes**		
	60	90	120
Class-1 (Stream)	91.66	100	100
Class-2 (Flower)	100	100	100
Class-3 (Jewel-Shaker)	100	100	100

Boldface data represents the best performance.

**Table 7:** **Confusion matrix**

**Class**	**Testing set**			**Validation set**		
	**C1**	**C2**	**C3**	**C1**	**C2**	**C3**
C1	64	0	0	16	0	0
C2	0	34	0	0	8	0
C3	0	0	30	0	0	8

**Figure 9:**
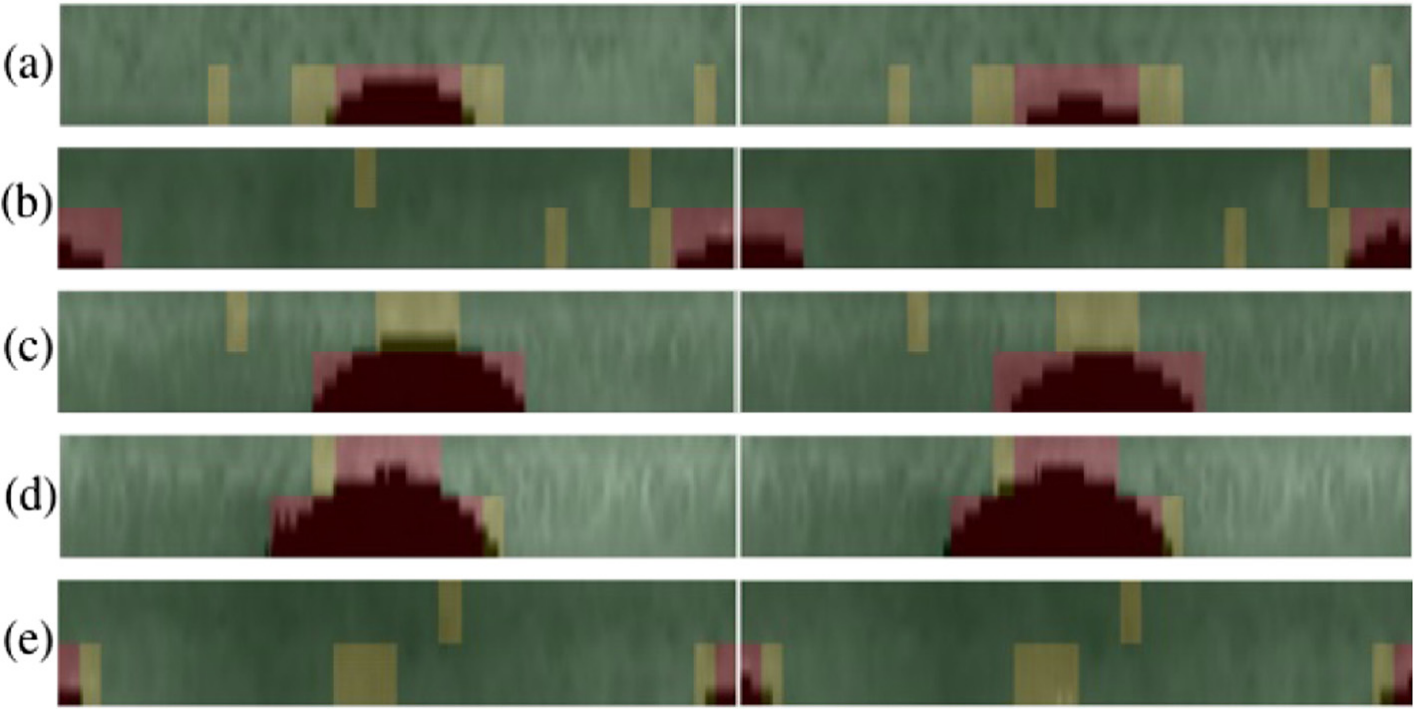
Iris adjudication: genuine iris matches with hamming distances **(a)** 0.21, **(b)** 0.19, **(c)** 0.16, **(d)** 0.15, **(e)** 0.19.

**Figure 10:**
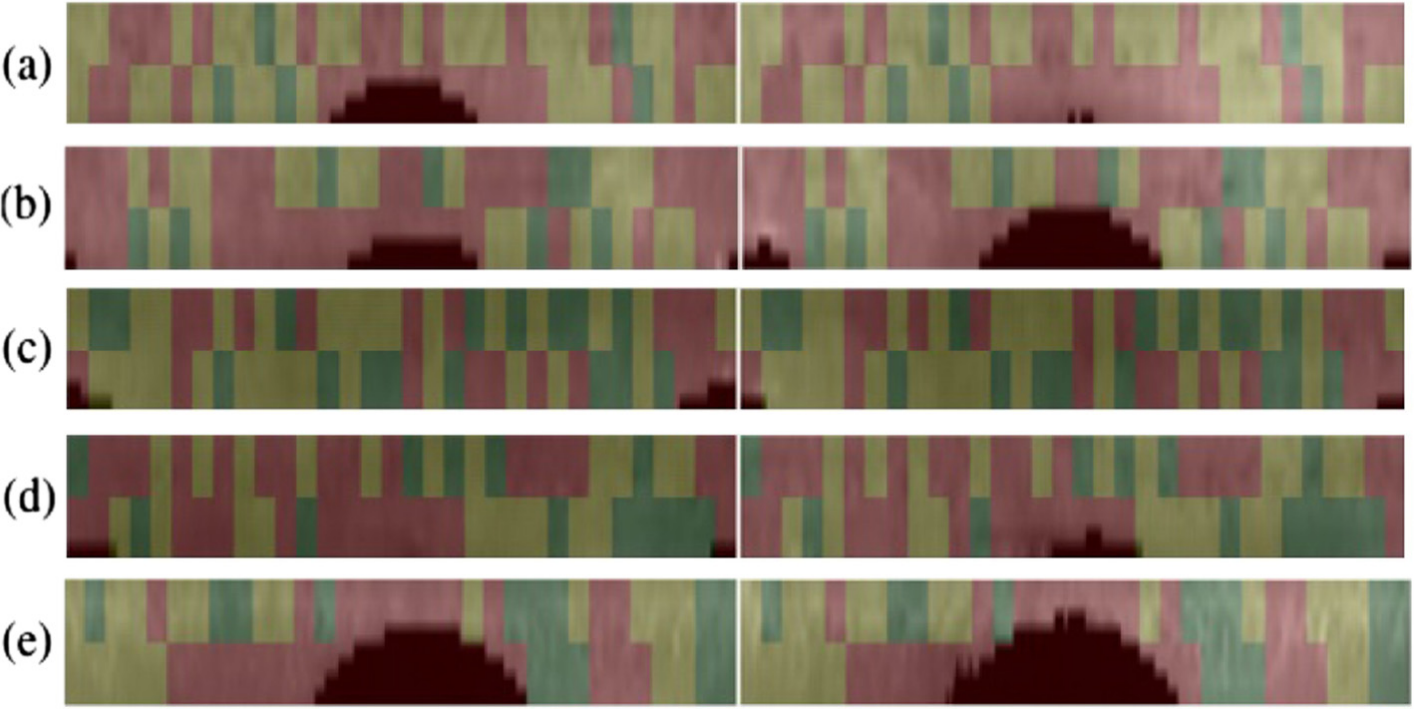
Iris adjudication: impostor iris matches with hamming distances **(a)** 0.48, **(b)** 0.46, **(c)** 0.43, **(d)** 0.51, **(e)** 0.37.

The adjudication results for genuine iris matches are illustrated in Figure 9 and for the impostor iris matches are given in Figure 10. The normalized images shown on these figures are taken from CASIA database for better illustration of adjudication process.

## Conclusion

In this paper, an iris classification is proposed based on sparse representation of log-gabor wavelet features using on-line dictionary learning (ODL) for large-scale de-duplication applications. Three different iris classes based on iris fiber structures, namely, stream, flower, jewel and shaker, are used for faster retrieval of identities. Also, an iris adjudication process is illustrated by comparing the matched iris-pair images side-by-side to make the decision on the identification score using color coding. The efficacy of the proposed classification approach is demonstrated on the standard iris database, UPOL, and it is achieved 100% classification accuracy with dictionary size 90 and residual error 0.05. The proposed iris de-duplication architecture improves the speed of identification process and reduces the identification errors in large-scale de-duplication applications.
